# sEMG-Based Hand-Gesture Classification Using a Generative Flow Model

**DOI:** 10.3390/s19081952

**Published:** 2019-04-25

**Authors:** Wentao Sun, Huaxin Liu, Rongyu Tang, Yiran Lang, Jiping He, Qiang Huang

**Affiliations:** 1 Key Laboratory of Biomimetic Robots and Systems, Ministry of Education, Beijing 100081, China; sun_wentao@outook.com (W.S.); qhuang@bit.edu.cn (Q.H.); 2 School of Mechatronical Engineering, Beijing Institute of Technology, Beijing 100081, China; JIPING.HE@asu.edu; 3 Beijing Innovation Centre for Intelligent Robots and Systems, Beijing 100081, China; langyiran@sina.com

**Keywords:** surface electromyography, hand-gesture classification, generative flow model

## Abstract

Conventional pattern-recognition algorithms for surface electromyography (sEMG)-based hand-gesture classification have difficulties in capturing the complexity and variability of sEMG. The deep structures of deep learning enable the method to learn high-level features of data to improve both accuracy and robustness of a classification. However, the features learned through deep learning are incomprehensible, and this issue has precluded the use of deep learning in clinical applications where model comprehension is required. In this paper, a generative flow model (GFM), which is a recent flourishing branch of deep learning, is used with a SoftMax classifier for hand-gesture classification. The proposed approach achieves 63.86±5.12% accuracy in classifying 53 different hand gestures from the NinaPro database 5. The distribution of all 53 hand gestures is modelled by the GFM, and each dimension of the feature learned by the GFM is comprehensible using the reverse flow of the GFM. Moreover, the feature appears to be related to muscle synergy to some extent.

## 1. Introduction

Surface electromyography (sEMG)-based hand-gesture classification is widely used in clinical applications, such as controlling powered upper-limb prostheses [1] and electric-powered wheelchairs [2]. However, sEMG is different from subject to subject, and even sEMG of the same subject can differ substantially due to the displacement of sensors, sweat, fatigue, and many other factors [3]. To capture the complexity and variability of sEMG, conventional pattern-recognition methods have been used to try to extract representative features of sEMG. Time-domain and frequency-domain features of sEMG, such as root mean square, zero crossing, and power spectra, have been widely used for hand-gesture classification [4]. However, these features are neither adequately generalizable to different subjects nor sufficiently robust for long-time applications [5]. To address these limitations, conventional approaches usually propose an easy-to-train model for each individual subject, and the model is frequently retrained once the performance of the model downgrades [6]. To avoid tedious retraining, more generalizable and robust sEMG features are needed to solve the problem of sEMG-based hand-gesture classification.

Prominent deep learning has improved the performance of solutions for many practical problems. The performance of deep learning lies in the ability to learn high-level abstraction of data and discover important information hidden in the data that is otherwise hard to discover using conventional algorithms [7]. Convolutional neural network(s) (CNN) are the most popular deep learning models used for sEMG-based hand-gesture classification [8,9,10], and state-of-the-art classification accuracy has been achieved using CNN. Other deep neural networks, such as Recurrent Neural Network(s) (RNN), have also been used for hand-gesture classification [11,12]. Most of the deep learning models used for sEMG-based hand-gesture classification are deep discriminative models that model the decision boundary between classes. Despite the high accuracy achieved with these deep discriminative models, the high-level features used in the deep discriminative models are incomprehensible. However, under some clinical conditions, model comprehension is crucial. For controlling prostheses, knowing the reason an algorithm that is performing well for one person with an amputation does not work for another person with an amputation is important for tuning the algorithm to fit different persons. Currently, comprehending the discriminative model is one of the research focuses of deep learning [13,14].

The deep generative model, which is a type of deep learning model other than the deep discriminative model, models the actual distribution of the data. A deep generative model can generate input examples from the feature learned by the model, which provides a way to understand the behavior of the model. However, deep generative model is usually used for applications such as fake image generation [15] or speech synthesis [16], and the model has no ability to discriminate input examples.

In this paper, a generative flow model (GFM) is used with a simple SoftMax classifier for hand-gesture classification. GFM is an unsupervised model commonly used for generating images [17] and synthesizing speeches [18]. In these cases, attention has been paid to the performance of the GFM in generating realistic samples, such as images and speeches. However, we focused on understanding the factorized features learned by the GFM and applying the learned features for supervised tasks. The combination of the GFM with a linear SoftMax classifier has achieved high accuracy in sEMG-based hand-gesture classification. In addition, the features learned by the GFM under the regulation of the SoftMax classifier have physiological relevance to the muscle synergy, which is important for comprehending the classification. The proposed approach achieved 63.86±5.12% accuracy in classifying 53 different hand gestures from the NinaPro database 5 [19]. Since the high-level feature learned by the GFM is factorized, each dimension of the feature was analyzed individually using the reverse flow of the GFM. Interestingly, each dimension of the learned feature was found to correspond to a basic sEMG pattern that may reflect human muscle synergy in the sEMG.

## 2. Physiology Background of Surface Electromyography

**Figure 1:**
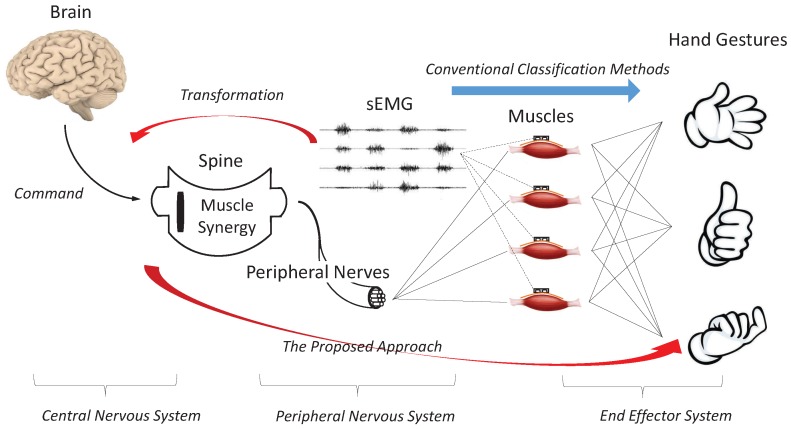
Human hierarchical system for controlling hand gestures.

Human hands are controlled by a hierarchical structure, as shown in Figure 1. This hierarchical structure can be divided into three systems according to their physiological properties. In the central nervous system, the movement command is generated from the brain to the spine. In the peripheral nervous system, the spine activates the corresponding muscles based on the command. Please note that the spine does not activate each muscle individually; instead, several muscles are activated simultaneously as a group to drive the hand to the target gesture. This mechanism of activating several muscles as a group is referred to as muscle synergy, a physiological process used by the human to control high-dimensional systems through low-dimension commands [20]. In the end effector system, the muscles drive the hand joints to the targeted position.

sEMG is the bio-electrical signal generated by the muscle when activated by the spine. Conventional classification methods map the sEMG to hand gestures directly, which tries to approximate the low-level end effector system. The proposed GFM transforms sEMG into factorized features, which are, ideally, an approximation of the movement command to the spine activating the mechanism of muscle synergy. Transforming sEMG into high-level abstractions can, to some extent, increase the generalizability and robustness of the following hand-gesture classification because high-level abstractions are believed to be more easily shared among different subjects than low-level information.

## 3. Methods

GFM is a recent flourishing branch of the deep generative model for transforming input data into meaningful features while requiring little or no human supervision or labeling. This model provides a practical way for employing deep learning for EMG-based hand-gesture classification and for comprehending the model. The transformed data by the GFM conform to a factorized distribution, which results in independent latent variables. Since such features are learned from unlabeled datasets and are not necessarily task-specific, downstream solutions based on the independent latent variables could potentially be more robust and more data efficient [21]. GFM requires fewer sEMG examples to learn by maximizing the exact log-likelihood of the input data compared to other widely used unsupervised deep learning approaches, such as DBN [22,23] and stacked AE [24]. These unsupervised approaches learn by maximizing the lower bound of the log-likelihood of the input data [25,26], which requires more training examples. The combination of a GFM with a simple classifier can leverage the advantages of deep learning for learning highly abstracted, robust features from the data. Meanwhile, the physiological meaning of the factorized sEMG feature can be analyzed by reversing the factorized feature to the sEMG through the reverse flow of GFM.

### 3.1. Surface Electromyography Signal Processing

**Figure 2:**
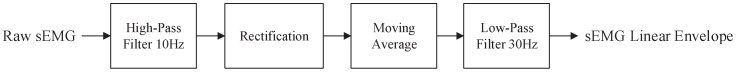
Pipeline for calculating sEMG linear envelope. Modified from [27] with permission.

The raw sEMG recorded by the two Myo armbands consists of 16 channels of sEMG sampled at a sampling rate of 200 Hz. Each channel of the raw sEMG is processed to obtain its linear envelope before being input to the GFM. The commonly used pipeline for calculating the sEMG linear envelope [27] is adopted as shown in Figure 2. The sEMG is high-pass filter with a cutoff at 10 Hz to remove movement artefacts. Then, the signal is rectified with an absolute value before being smoothed with a moving average of 10 sampling points. Finally, the signal is low-pass filtered at 30 Hz to obtain the envelope signal. The multi-channel sEMG envelope is segmented with 64 sampling points. Thus, the input sEMG envelope for the GFM has the size 64×16.

### 3.2. Generative Flow Model

The starting point of GFM involves estimating an unknown distribution $p_{x}\left(x\right)$ by a simple factorized known distribution $p_{z}\left(z\right)$ given a dataset of $x=[x_{1},\ldots ,x_{N}]$ sampled from the unknown distribution. The generative flow model defines a parametric invertible transformation function $f_{\theta }\left(x\right):x\to z$ that directly maps the input data *x* into the known prior *z* [28]. Normally, a spherical multivariate Gaussian distribution $p_{z}\left(z\right)=N\left(z;0,I\right)$ is used as the known prior. The log probability distribution of a sample $x_{i}$ from the given dataset *x* according to the change in the variable formula mentioned in [29] is calculated as:

(1) $$\log p_{x}\left(x_{i}\right)=\log p_{z}\left(f_{\theta }\left(x_{i}\right)\right)+\log det\left(\frac{\partial f_{\theta }\left(x_{i}\right)}{\partial x_{i}}\right)$$

The training of the θ parameters is for learning a continuous nonlinear transformation *f* that is differentiable almost anywhere to maximize the log-likelihood of the whole given dataset *x* [29]. For ease of training, GFM requires the determinants of the Jacobian matrix of the transformation function *f* to be tractable. We use a combination of the four modules actnorm, invertible 1 × 1 convolution, affine coupling layer, and multi-scale architecture as suggested in [21,30] to construct the transformation *f*. The determinants of the Jacobian matrices of the first three modules mentioned are simply multiplications of their diagonal elements. The multi-scale architecture pushes half of its input to conform to a Gaussian distribution, which can reduce the variability of the latent variables caused by noise. Details on the transformation, its reverse flow, and the log-determinants of each module are shown in the Appendix A. The proposed GFM is composed of 3 flow steps, and the first two steps consist of a sequence including an actnorm, an invertible 1 × 1 convolution, an affine coupling layer, and a multi-scale architecture, while the last flow step is composed of the same sequence except for the multi-scale architecture. The multi-scale architecture divides the dimension of its input by 2. The input sEMG linear envelope for the GFM has a size of 64×16, and the output of the factorized feature has a size of 32×8.

### 3.3. Classifier

GFM is an unsupervised learning approach. To use the factorized feature in supervised tasks, such as hand-gesture classification, GFM is combined with a SoftMax classifier. Since the input to the classifier is the factorized feature, a simple linear SoftMax classifier is adopted. To train the classifier in conjunction with GFM, the parameters θ of the GFM and the parameters ϕ of the classifier are updated simultaneously to minimize the negative log-likelihood of the given dataset *x* and the cross-entropy loss between the output of the classifier $\hat{y}$ and the movement label *y*. The compound objective function modified from the original objective function of GFM [21] is:

(2) $$\begin{array}{cc} L & =\underset{\theta ,\;\phi }{\arg\min}\left\{-\sum_{N}\left[\log p_{z}\left(f_{\theta }\left(x_{i}\right)\right)+\log det\left(\frac{\partial f_{\theta }\left(x_{i}\right)}{\partial x_{i}}\right)\right]+\;CE\left(\hat{y},y\right)\right\}\\ \;\hat{y} & =c_{\phi }\left(f_{\theta }\left(x\right)\right) \end{array}$$

where *L* is the compound objective function, *c* stands for the SoftMax classifier, and CE is the cross-entropy loss. With the cross-entropy loss of the classifier included in the objective function, the distributions of the factorized feature *z* will be regulated by the classifier. Accordingly, the label information is used to train the transformation *f*.

**Figure 3:**
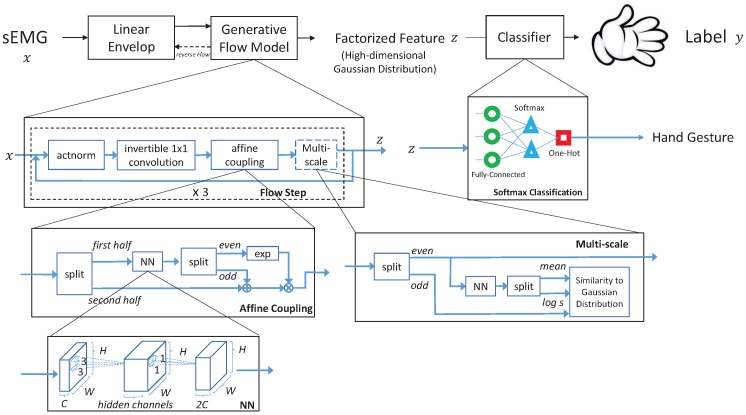
Overall structure of the proposed approach.

The overall structure of the proposed approach is shown in Figure 3. Output from the GFM is fed to the classifier for classifying hand gestures. The combination of the GFM with a classifier is not simply concatenating the output of the GFM to the input of the classifier, but the two parts are trained together. The last term of Equation (2) changes the original objective function of GFM, which allows the GFM and the classifier to interact with each other in the training.

## 4. Experiment

**Figure 4:**
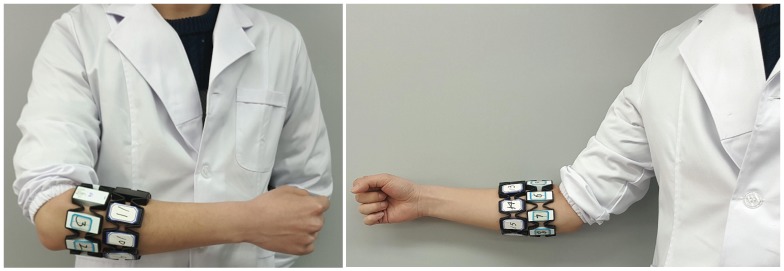
Acquisition setups for the two Myo armbands.

To test the performance of the proposed approach in hand-gesture classification, the proposed model is trained with the NinaPro database 5, which contains sEMG recorded with two Myo armbands. sEMG from the Myo armbands are sampled at a rate of 200 Hz. The two Myo armbands, each including 8 active single differential wireless electrodes, are placed next to each other, as shown in Figure 4. The top Myo armband is placed close to the elbow with the first sensor placed on the radio humeral joint; the second Myo armband is placed just after the first, near the hand, and is tilted by 22.5 degrees with respect to the first Myo armband. Database 5 includes 6 repetitions of 53 different movements (shown in the Appendix A) performed by 10 intact subjects. Please note that the number of sEMG examples for hand rest is much larger compared to the other movements, and we randomly sampled some of the rest sEMG examples to guarantee a balance in the sEMG examples for different hand gestures.

Two methods are used to divide the NinaPro database 5 into a train set and test set. In the first approach, the database is divided in a machine-learning way: 70% of the sEMG examples are randomly selected from the database and used to train the model, while the remaining 30% of the sEMG examples are used to test the accuracy of the proposed approach in classifying hand gestures. In the second approach, the database is divided according to subjects: sEMG examples of 7 randomly selected subjects are used to train the model, while sEMG examples of the remaining 3 subjects are used as a test set. The main difference between these two methods of division is whether the model can see sEMG examples from a subject in both the training and test sets. Obviously, the second division approach better matches the real-world applications for sEMG. In both cases, the proposed model was trained for 15 epochs at a batch size of 24. The average accuracy of the proposed approach was achieved by repeating the training and testing procedures 5 times.

## 5. Results

**Figure 5:**
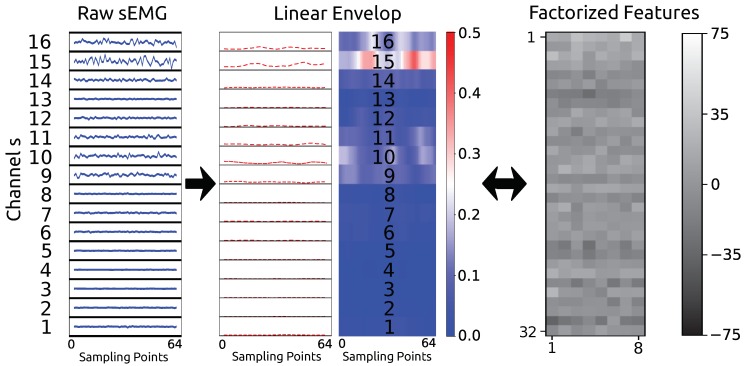
Work flow for the proposed approach.

The raw sEMG data were transformed into factorized features, as shown in Figure 5, using the linear envelope pipeline and GFM. Please note that the transformation between the linear envelope and the factorized feature is invertible.

**Figure 6:**
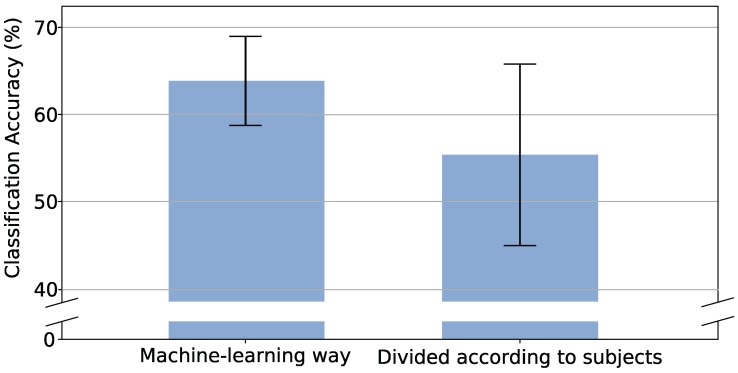
Hand-gesture classification accuracy for different test sets.

As shown in Figure 6, the classification accuracy of the proposed approach on the test set divided according to subjects is 55.37%±10.43%. In addition, the classification accuracy of the proposed approach on the test set divided in the machine-way, where sEMG examples of the test set are randomly selected from the NinaPro database 5, is 63.86%±5.12%. The proposed approach, as expected, performs worse on the test set divided according to subjects than the test set divided in the machine-learning way. This performance reduction is caused by the approach lacking enough knowledge about the sEMG examples of a specific subject. However, the reduction is only 8.49%, which means that the proposed approach has learned some common features across subjects.

**Figure 7:**
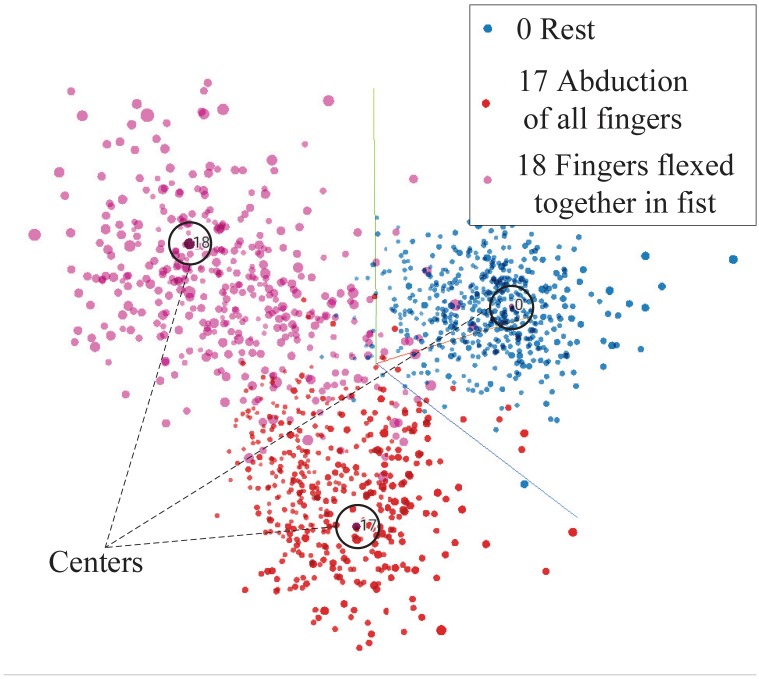
Distributions of hand rest, hand open, and hand close in the feature space. Total variance described: 24.5%

GFM, as a generative model, models the actual distribution of the data based on the distribution of the factorized feature. To see the distribution of the factorized feature and its association with different hand gestures, three commonly used hand gestures for prosthetic hand control are selected, and the distribution of their factorized features is shown in Figure 7. The figure is a snapshot from TensorBoard [31]. The distribution of the features of all 53 hand gestures is available in the Appendix A. From Figure 7, we can see that features belonging to the same hand gesture are gathered, and the features of the three gestures are perfectly distinguishable. Since each dimension of the feature is continuous and independent, features corresponding to a hand gesture are summed and averaged to determine the center of the hand gesture. The center of a hand gesture is also considered the typical feature of the hand gesture.

**Figure 8:**
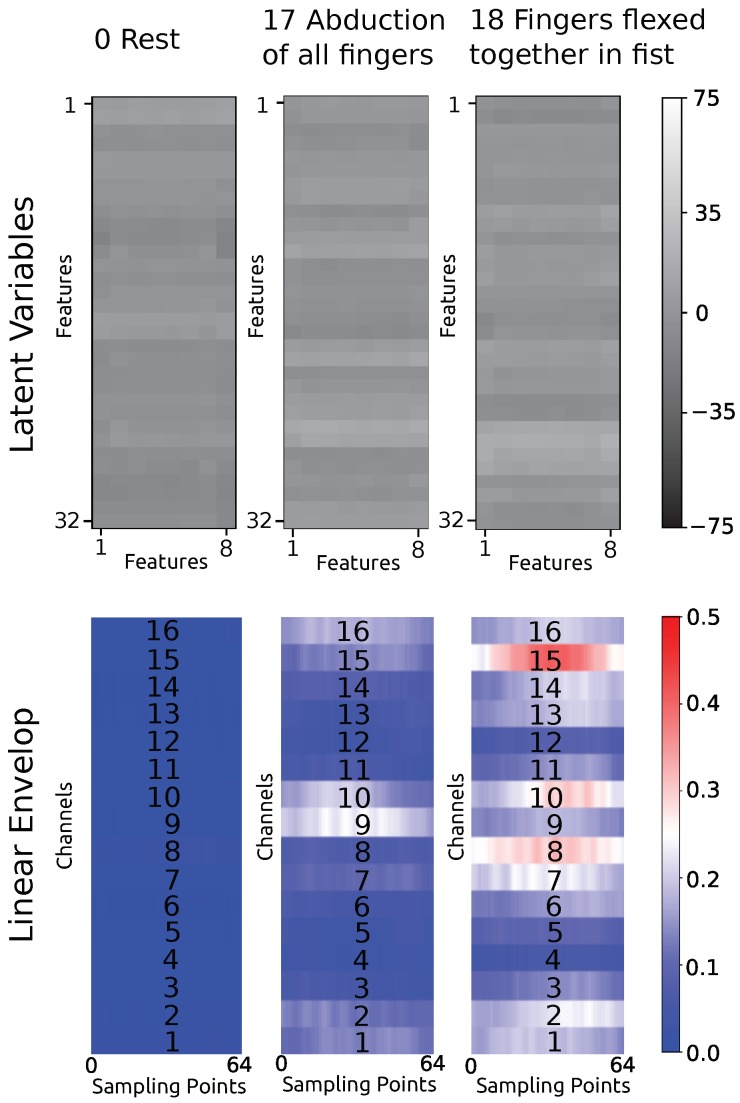
The feature centers of different hand gestures, and their reverse transformation to the sEMG linear envelope.

The typical features of the three hand gestures are transformed by the reverse flow of the GFM to the corresponding sEMG linear envelope. Features and the transformed sEMG linear envelope corresponding to hand gesture 0 (rest), 17 (abduction of all fingers), and 18 (fingers flexed together in fist) are shown in Figure 8. We can see that the typical feature of rest is transformed to a zero sEMG linear envelope, which correlates with the rest movement. Furthermore, hand gesture 17 and 18 differ mainly in the activation of channel 8 and 15. Channel 8 and 15 cover the activity of the flexor carpi ulnaris muscle of the forearm, which acts to flex and adduct the hand. Physiologically, the activity of the flexor carpi ulnaris muscle is useful for distinguishing hand opening and hand closing.

**Figure 9:**
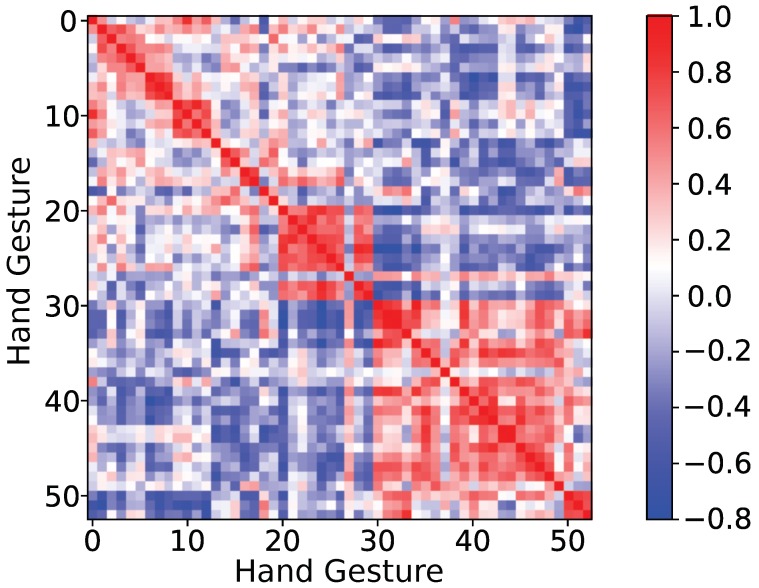
Correlation matrix for the proposed approach.

The correlation matrix is important for evaluating the ability of a generative model in distinguishing different classes. Each element of the correlation matrix is calculated as the cosine of the angle between two hand-gesture centers. A hand-gesture center can be treated as a vector connecting the origin of coordinates to the center. The cosine of the angle between two vectors evaluates their correlations. The correlation matrix of the proposed approach is shown in Figure 9. If the correlation between two hand-gesture centers is close to 1, then the two hand gestures are hard to distinguish from each other.

**Figure 10:**
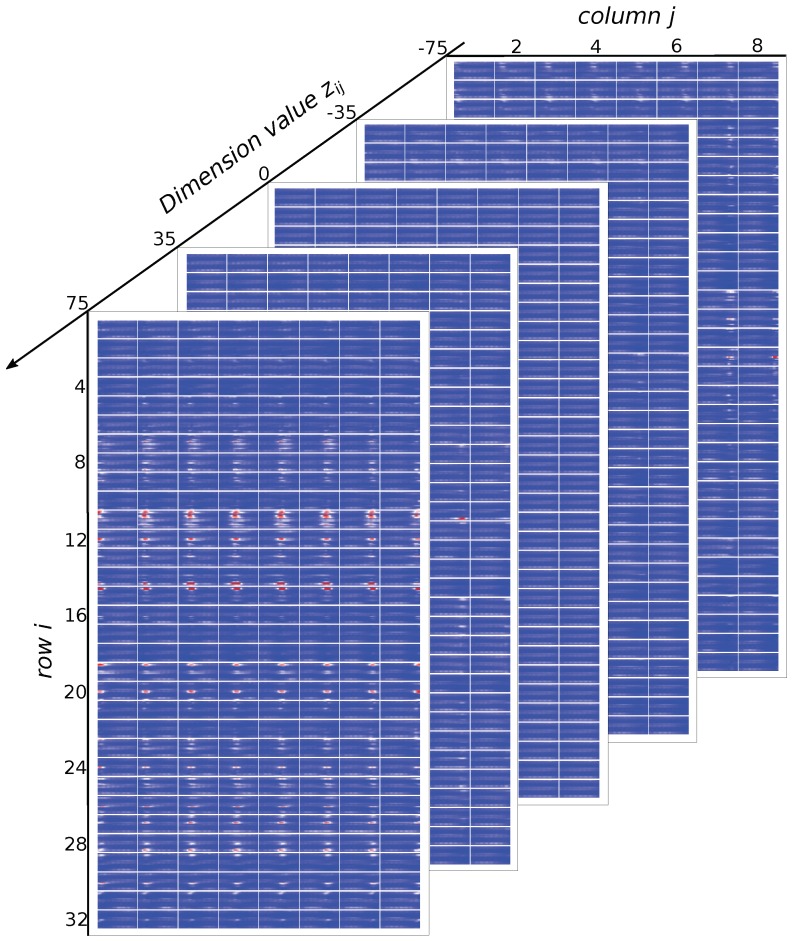
The sEMG linear envelope corresponding to each dimension of the factorized feature.

Since the feature learned by the GFM is factorized, each dimension of the feature is analyzed individually to evaluate its relation to the sEMG linear envelope. Figure 10 shows sub-images arranged along the axis of dimension value z in 32 rows and 8 columns in which the sub-image at row *a* and column *b* is generated by a factorized feature with $\;z_{i,j}=v,\;if\;i=a\;and\;j=b,otherwise,\;z_{i,j}=0;\;v\in \left[-75,75\right],\;i=1,2,\ldots ,32,\;j=1,2,\ldots ,8$. The dimension value *v* is constrained in [-75,75] because ±75 is the minimum/maximum value of the feature obtained in the train set, and a dimension with value >75 or <−75 is the output of the reasonable range of the feature. In Figure 10, generated sEMG linear envelopes corresponding to some selected *v* values v=[-75,-35,0,35,75] are shown. From the results, we can see that *v* determines the strength of the generated sEMG linear envelope, and the strength of the sEMG linear envelope increases with increasing *v*. We can also determine that each row of the factorized feature corresponds to a basic pattern of the sEMG linear envelope, which may reflect the muscle synergy taking effect underneath the sEMG recording channels. Each column of the feature corresponds to the spreading of the basic pattern with time. For example, the 11th row corresponds to a pattern of the sEMG linear envelope, where channels 16, 15, 12, 11, 10, and 4 are activated simultaneously, and the columns determine the occurrence time of the pattern. At the first column, the pattern occurs on the left side of the sub-image, while at the 8th column, the pattern occurs at the right side of the sub-image. The left/right side of the sub-image corresponds to the occurrence of a pattern in the sEMG linear envelope. In summary, the 32×8 factorized feature is interpreted as follows: the 32 rows determine 32 different sEMG linear envelope patterns, the 8 columns determine the occurrence of the patterns in the sEMG linear envelope, and the elements of the matrix determine the strength of the sEMG linear envelope.

We made a GUI for the readers to interact with the proposed approach to see the relation between the factorized feature *z* and the corresponding sEMG linear envelope. The GUI is available in the Appendix A.

## 6. Discussion

A deep learning approach that can extract comprehensible features from sEMG for hand-gesture classification was proposed. The approach allows employing deep learning to clinical applications of sEMG-based hand-gesture classification where model comprehension is required.

As a newly coming approach, GFM has not been widely used in applications other than image generation and speech synthesis. In most cases, the Gaussian-distributed factorized features learned by GFM are uninterpretable. Since images and speeches are intuitive for human, one can manually interpret the meaning of the factorized feature by tuning the factorized feature and check its influences on the generated samples from GFM. However, for biomedical signal, it is difficult to interpret the meaning of the factorized feature without a task-related label/event to check the generated biomedical samples from GFM. In the paper, a well-designed GFM trained under regulation of a linear SoftMax classifier can learn good features for classification, and the task-related features are well-interpretable.

The proposed approach has achieved competitive accuracy for classifying hand gestures of NinaPro database 5 compared with existing methods. Overall, 69.04% ± 5.24% accuracy for 41 selected hand gestures from the database was achieved by the method described in [19] using the support vector machine (SVM) algorithm and multivariate discrete wavelet technique (mDWT). The sEMG used in the method was segmented at a window of 200 sampling points with an overlap of 100 sampling points. Meanwhile, 82.15% accuracy was achieved by a CNN described in [32] for 17 selected hand gestures; however, the CNN required pre-training using sEMG from other databases. The sEMG used in this method was segmented at a window of 16 sampling points. In the paper, NinaPro database 5 was chosen for comparing the hand-gesture classification accuracy of the proposed approach with other existing algorithms. In addition, using an open-source database is good for other researchers to verify the results. Since the proposed approach is applicable to most of the EMG databases by designing a proper input/output flow of GFM and a linear classifier, we will be interested in applying the approach to robotic prosthesis control in the future.

In designing the classifier, the linear SoftMax classifier used in the proposed approach finds a good balance between learning a good distribution of the factorized feature and achieving a high classification accuracy. Actually, we had tried to combine GFM with nonlinear classifiers, such as SoftMax classifier with multiple hidden layers and CNNs. Although the nonlinear classifiers can increase the classification accuracy a little bit, the factorized feature learned by the GFM under the regulation of the nonlinear classifiers is incomprehensible. The distribution of the factorized features belonging to a hand gesture is not gathered as shown in Figure 7, but the features are scattered around. The scattered features have little physiological meaning and are uninterpretable.

The red square at the right bottom of the correlation matrix suggests that it is hard to distinguish hand gestures 30∼52. Hand gestures 30∼52 belong to exercise C, which consists of human grasping and functional movements. The difficulty in distinguishing functional movements is caused by the fact that functional movements activate most of the forearm muscles, while the isometric movements in exercise A and B only activate a small portion of the forearm muscles. With more muscles recruited, the hand can both exert large force and reduce fatigue while performing function movements. Based on the correlation matrix, the remarkable hand gestures out of the 53 hand gestures can be selected to reduce improper classification. Most of the widely used discriminative methods lack a way to calculate their correlation matrix for hand gestures because instead of modelling the actual distribution of the hand gestures as the proposed approach, these methods only model the decision boundary of the hand gestures, which removes the correlation information.

The analysis of each dimension of the factorized feature indicates that its rows correspond to different sEMG linear envelope patterns, its columns correspond to the occurrence of the patterns, and its elements determine the strength of the pattern. The regular pattern of the factorized feature is more comprehensive than we expected. Often, representations of features in deep learning models are hard for humans to understand. Since the proposed approach is trained to distinguish hand gestures of different subjects, muscle synergy, which is a common mechanism shared among subjects, is theoretically an optimal feature for the task. From the results, we can see that the proposed approach learned to represent the sEMG linear envelope as a combination of some basic patterns of the sEMG. We suppose that these basic patterns may be reflections of the muscle synergy in the sEMG. However, the dimension of the factorized feature is much larger than that of muscle synergy, which means that there are some redundancies in the factorized feature. The dimension of the factorized feature can be reduced by adding more multi-scale modules to the GFM. However, with the dimension of the feature reduced, the accuracy of the classifier may decrease.

## 7. Conclusions

This study has developed a comprehensible deep learning model for sEMG-based hand-gesture classification. The developed model allows the use of deep learning in clinical applications for which model comprehension is required. This study serves as a basis for future studies on employing deep learning in sEMG-based applications. Although the approach analyzes each individual dimension of the factorized feature, the exact relation between the feature and the muscles is not fully understood. In the study, we assumed that the feature reflects the muscle synergy. Further research needs to be conducted to examine more closely the links between the factorized feature and the muscle synergy.

## Figures and Tables

**Figure A1:**
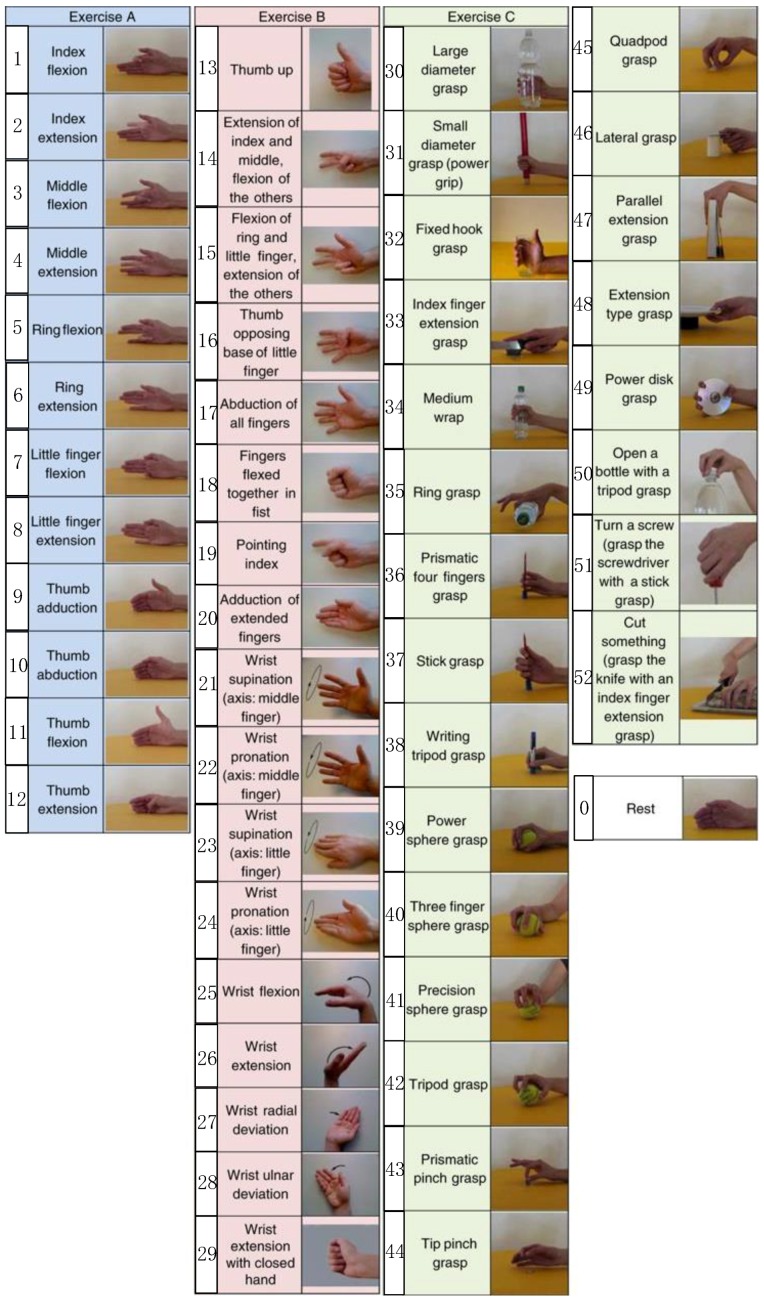
Hand gestures of the NinaPro database. Modified from [34] licensed under a Creative Commons Attribution 4.0 International License.

**Figure A2:**
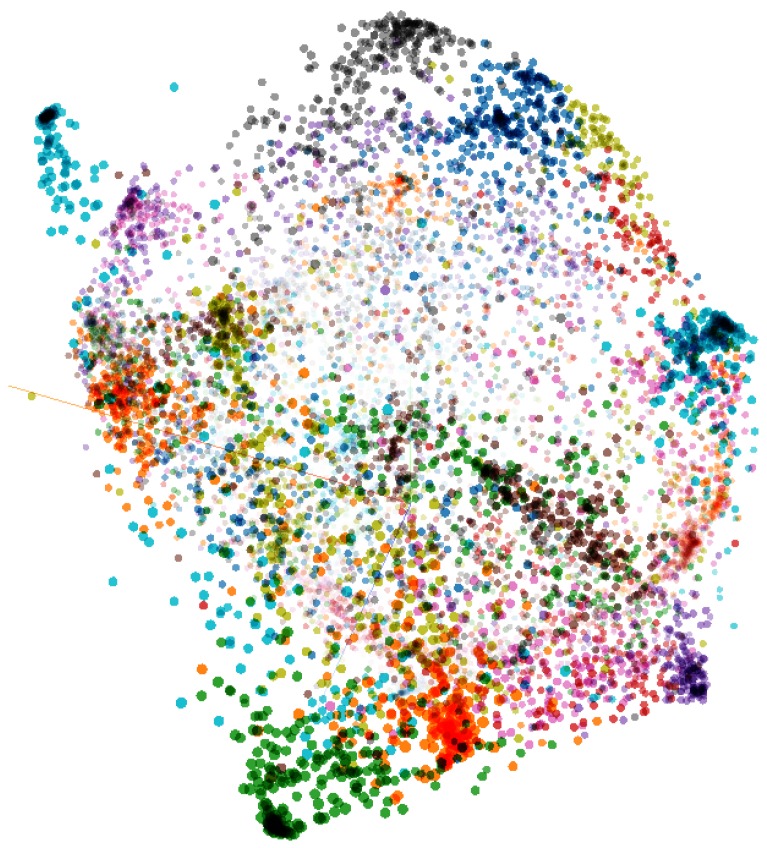
Distribution of all 53 hand gestures (labeled by colors) in the latent variable space.

**Figure A3:**
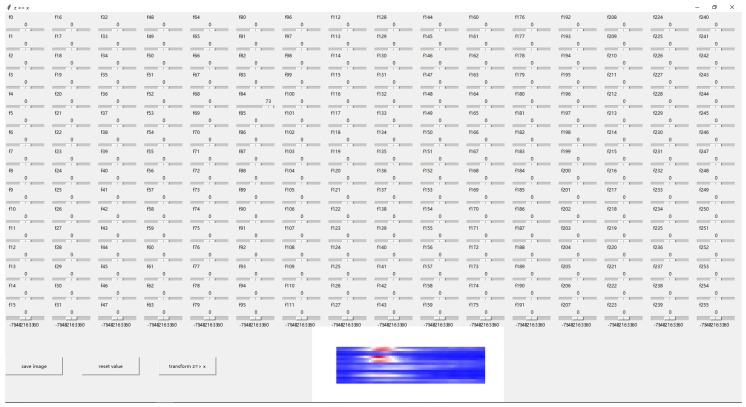
A GUI for interacting with the proposed approach. The fi;i=0∼255 denotes the i+1-th dimension of the factorized feature. The transform button transforms the current feature to the sEMG linear envelope and shows the result on the canvas at the bottom. The reset button is used to save the canvas. The reset button is used to set all the dimensions of the feature to 0.

## Abbreviations

The following abbreviations are used in this manuscript:

sEMG	Surface electromyography signal
GFM	Generative flow model
CNN	Convolutional neural network
RNN	Recurrent neural network
AE	Auto-encoder
DBN	Deep belief network

## Appendix A

### Appendix A.1. Modules Used in the Generative Flow Model

In total four modules, Actnorm, Invertible 1 × 1 convolution, Affine coupling, and multi-scale are used in the GFM. The transformation, its reverse flow and the log-determinant of the first three modules are similar to those mentioned in [21]. The transformation of the multi-scale module is calculated as:

(A1) $$\begin{array}{cc} x_{a},x_{b} & =split(x)\\ mean,\log s & =\mathrm{NN}(x_{a})\\ y & =x_{a} \end{array}$$

where *x* signifies the input of the multi-scale module, *y* signifies the output of the multi-scale module. Both *x* and *y* are tensors of shape [h×w×c] with spatial dimension (h,w) and channel dimension *c*. split(x) is a function that splits the tensor *x* into two tensors along the channel dimension by half or by parity (in even and odd order). NN is a CNN with a hidden layer.

The reverse flow of the multi-scale is calculated as:

(A2) $$\begin{array}{cc} mean,\log s & =\mathrm{NN}(y)\\ x_{b} & =N(mean,s^{2})\\ x_{a} & =y\\ x & =concat(x_{a},x_{b}) \end{array}$$

where N denotes the Gaussian distribution and concat() is a function that concatenates two tensors along the channel dimension.

The log-determinant of the multi-scale is calculated as:

(A3) $$logdet=-\frac{1}{2}\left[2\log s+\frac{{\left(x_{b}-mean\right)}^{2}}{e^{2\log s}}+\log2\pi \right]$$

### Appendix A.2. Code for the Proposed Approach

The code for dividing the NinaPro database 5 is available at https://github.com/sun2009ban/divide_NinaPro_database_5.

The code for visualizing the distribution of the 53 hand gestures in TensorBoard is available at https://github.com/sun2009ban/tensorboard_53_hand_gestures.

The GUI for playing with the factorized feature of the model is available at https://github.com/sun2009ban/glow-pytorch-with-gui.

### Appendix A.3. Figures of the Hand Gestures and the GUI

Figure A1 shows 53 hand gestures in the NinaPro database 5. Figure A2 shows the distribution of the 53 hand gestures in the latent variable space. The figure was drawn in TensorBoard using t-SNE method [33] for dimension reduction. Figure A3 presents a snapshot of the GUI for playing with the factorized feature. The horizontal scale bar corresponds to each dimension value of the factorized feature. The dimension value can be set [−75, 75]. By pressing the ‘transform z => x’ button, the sEMG linear envelope will be generated by the factorized feature determined by the scale bars using the inverse flow of the GFM, and the generated sEMG linear envelope will be shown on the canvas. Pressing the ‘reset’ button will reset all the dimensions of the factorized feature to zero. Pressing the ‘save image’ button will save the sEMG linear envelope shown on the canvas.
